# Preparation and characterization of novel double-decker rare-earth phthalocyanines substituted with 5-bromo-2-thienyl groups

**DOI:** 10.1186/s13065-017-0260-x

**Published:** 2017-04-05

**Authors:** Jiří Černý, Lenka Dokládalová, Petra Horáková, Antonín Lyčka, Tomáš Mikysek, Filip Bureš

**Affiliations:** 1 Centre of Organic Chemistry Ltd., Rybitví 296, 53354 Rybitví, Czech Republic; 2 Department of Analytical Chemistry, University of Pardubice, Faculty of Chemical Technology, Studentská 573, 53210 Pardubice, Czech Republic; 3 Institute of Organic Chemistry and Technology, University of Pardubice, Faculty of Chemical Technology, Studentská 573, 53210 Pardubice, Czech Republic

**Keywords:** Rare-earth bisphthalocyanines, UV–vis spectroscopy, NIR spectroscopy, Singlet oxygen production, Reduction, Cyclic voltammetry, Acid stability, Thermogravimetry

## Abstract

**Background:**

A series of rare-earth bisphthalocyanines of praseodymium, samarium and gadolinium bearing 5-bromo-2-thienyl substituents were prepared for the first time.

**Results:**

Three bis[octakis(5-bromo-2-thienyl)] rare-earth metal(III) bisphthalocyanine complexes (Pr, Sm, Gd) were synthesized for the first time. The new compounds were characterized by UV–vis, NIR, FT-IR, mass spectroscopy and thermogravimetry as well as elementary analysis and electrochemistry. Production of singlet oxygen was also estimated using 9,10-dimethylanthracene method.

**Conclusions:**

The bromine substituent causes significant changes in molecule paramagnetism, singlet oxygen production, HOMO position and spectral characteristics. The compounds in solutions exist in two forms (neutral and/or reduced) depending on the solvent and rare-earth metal. Moreover, the compounds exhibit much increased stability under acid conditions compared with non-brominated derivatives.

**Graphical abstract:**
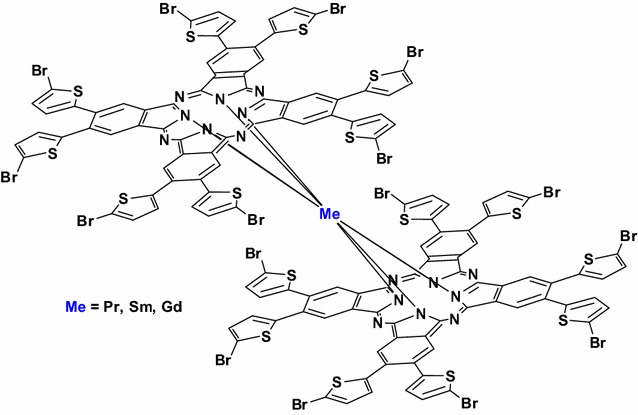
Prepared compounds for the study of their chemical and other properties.

**Electronic supplementary material:**

The online version of this article (doi:10.1186/s13065-017-0260-x) contains supplementary material, which is available to authorized users.

## Background

Double-decker rare-earth phthalocyanines were firstly reported by Kirin [1] in 1965. Since then, they found a lot of applications. Among them are colour and electrochromic displays [2], gas sensors [3], field-effect transistors [4] and nonlinear optical materials [5]. Widely studied are also their magnetic [6] and conducting properties [7]. For these applications, many unsubstituted and substituted derivatives were prepared and evaluated to date. Thiophene moieties as strong donors are very often adopted for tailoring electronic properties of many classes of compound studied for applications in organic electronics [8]. Recently, a series of three thiophene-substituted rare-earth bisphthalocyanines of gadolinium, praseodymium and samarium were studied by our group [9]. It was found that the compounds were very sensitive to the presence of an acid yielding metal-free phthalocyanines irreversibly. This unexpected instability can limit their use for organic electronics. Our working hypothesis was that the acid stability should be increased if suitable group is attached to the 2-position on the thiophene cycle. For this purpose, a bromo substituent was introduced to the phthalocyanine scaffold. The aim of this study was to evaluate the effect of this modification on their physical, photo-physical and electrochemical properties.

## Experimental

### General

All starting materials were obtained from Aldrich and Penta, and were used without further purification. Unsubstituted phthalocyanines were prepared according to the literature procedure [1].

The ultraviolet–visible (UV–vis) spectra were measured within the range of 300–900 nm on a UNICAM UV/VISIBLE Spectrophotometer, Helios Beta. The near infra-red (NIR) spectra were measured within 800–2100 nm on a PerkinElmer Lambda 1050 UV/VIS/NIR spectrometer. FT-IR spectra were recorded on a Nicolet 6700 FT-IR spectrometer. Thermogravimetric analyses were performed using a Mettler Toledo TGA/DSC 1 STARe System in a 70 ll alumina crucible. A small amount of the test compound (6–7 mg) was weighed into the measuring crucible and heated using a controlled temperature program between 25 and 700 °C using a gradient of 10 °C min^−1^. A flow of nitrogen (about 20 ml min^−1^) was used as a protective gas. During the heating process weight-curves were recorded over the complete temperature range. Elemental analyses were obtained using a FISONS EA 1108 automatic analyser. Matrix-assisted laser desorption/ionization time-of-flight mass spectra (MALDI-TOF) were measured on a MALDI mass spectrometer LTQ Orbitrap XL equipped with nitrogen laser. Positive-ion and linear mode of the compounds were obtained in trans-2-[3-(4-*tert*-butylphenyl)-2-methyl-2-propenylidene]malononitrile matrix for **2** and **3** and 2,5-dihydroxybenzoic acid matrix for **4** using nitrogen laser accumulating 10 laser shots. Electrochemical measurements were carried out in 1,2-dichloroethane containing 0.1 M Bu_4_NPF_6_. Cyclic voltammetry (CV) and rotating disk voltammetry (RDV) were used in a three electrode arrangement. The working electrode was platinum disk (2 mm in diameter) for CV and RDV experiments. As the reference and auxiliary electrodes were used saturated calomel electrode (SCE) separated by a bridge filled with supporting electrolyte and a Pt wire, respectively. All potentials are given vs. SCE. Voltammetric measurements were performed using a potentiostat PGSTAT 128N (Metrohm Autolab B.V., Utrecht, The Netherlands) operated via NOVA 1.11 software.

### Preparation of bis[octakis-(5-bromo-2-thienyl)phthalocyaninato] rare-earth metal(III) phthalocyanines (**2**–**4**)

**Scheme 1:**
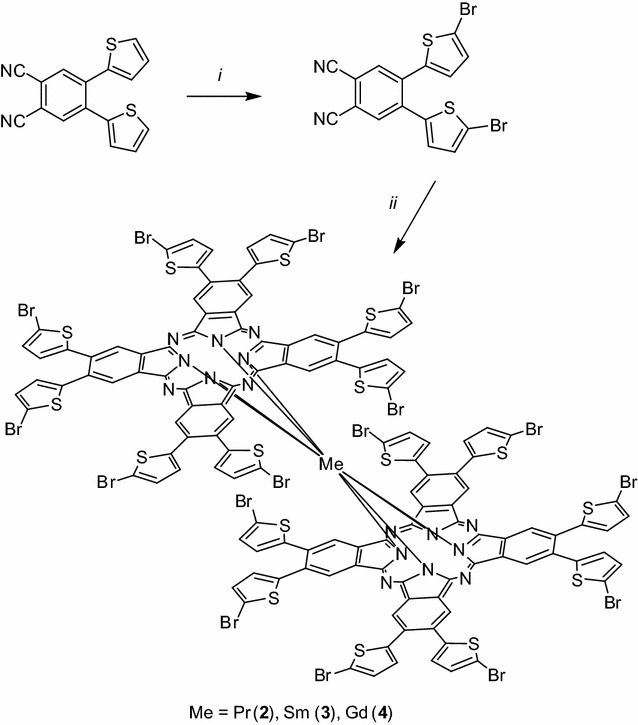
Synthesis of the starting nitrile 1 and rare-earth metal bisphthalocyanines **2**–**4**. Reagents and conditions: (*i*) *N*-bromosuccinimide, DMF, 0–25 °C, 65%. (*ii*) 1. Li, *n*-pentanol, 3 h, 135 °C, 2. (CH_3_COO)_3_Me, DMF, 10 h, 140 °C, **2**—Me = Pr 29%, **3**—Me = Sm 16%, **4**—Me = Gd 34%

The starting 4,5-bis(5-bromo-2-thienyl)phthalonitrile (**1**) was prepared by bromination of 4,5-bis(2-thienyl)phthalonitrile using *N*-bromosuccinimide in good yield. All the investigated bisphthalocyanines were synthesized from **1** by a two-step, one-pot reaction (Scheme 1). In the first step, the starting nitrile **1** was refluxed in *n*-pentanol with metal lithium under nitrogen. The resulting dilithium phthalocyanine was without isolation reacted with anhydrous rare-earth metal acetate dissolved in anhydrous DMF under reflux. The products were purified by flash chromatography using cellulose as the adsorbent and eluted first with ethyl-acetate and then with THF. The yields of pure **2**–**4** were 16–34%. Synthetic procedures including basic characterizations are given in Additional files 1 and 2.

## Results and discussion

### Characterization

The synthesized complexes **2**–**4** were characterized by several spectroscopic techniques—UV–vis, NIR, FT-IR, MALDI-TOF, thermogravimetry and elemental analysis. Proton NMR were measured in CDCl_3_ or THF-*d*_8_. No analysable signals were obtained, even by using a published trick [10] with oxidation with a large excess of bromine. The reduced forms (after addition of NaBH_4_ in THF-*d*_8_) also showed paramagnetism.

In these sandwiches (neutral compounds), one phthalocyanine ring is the classical dianion and the second one is the radical anion with charge −1. With a trivalent rare-earth metal cation, they form a neutral compound. Generally, in solutions they exist in two forms—a neutral and a reduced form. The distribution depends (Additional file 3) on the polarity and basicity of the solvent. The exact form in solutions are discussed in respective sections of the article.

### UV–vis spectral characteristics

**Fig. 1:**
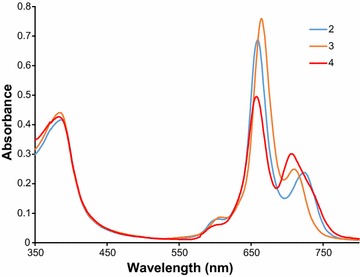
UV–vis spectra of rare-earth bisphthalocyanines *2*–*4* in DMF at 10 mg l^−1^

UV–vis spectra of **2**–**4** in DMF are presented in Fig. 1. They show typical features for bisphthalocyanines—a Soret band appearing at ca. 385 nm and two Q-bands, one located at wavelength of about 660 nm and the other at 710–720 nm. This is in agreement with reported spectral behaviour for octa-2,2,3,3-tetrafluoropropoxy rare-earth phthalocyanines [11] and it corresponds to reduced forms of bisphthalocyanines.

**Fig. 2:**
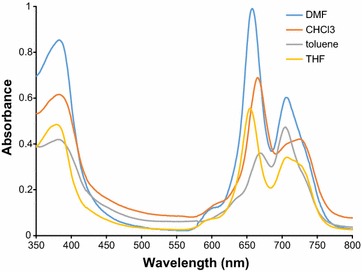
UV–vis spectra of 4 in polar and non-polar solvents (20 mg l^−1^)

UV–vis spectra of **4** in THF, toluene, DMF and CHCl_3_ are shown in Fig. 2. In THF and toluene is present an additional peak at ~700 nm (more pronounced for toluene). This peak is characteristic of a neutral form. Also, a new broad band appeared in 500–600 nm wavelength area. It corresponds to π-radical cation of the complex. Similar spectra were obtained for **2** and **3** (Additional file 3).

**Fig. 3:**
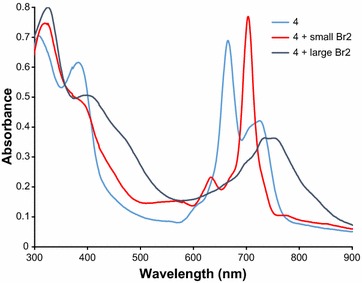
UV–vis spectra of 4 in CHCl_3_ at 20 mg l^−1^ upon addition of various amounts of Br_2_. *Red line* addition of 10 μl 0.01 M Br_2_, *black line* addition of 20 μl 0.44 M Br_2_

Figure 3 shows a typical change in the shape of spectra upon oxidation of **4** with bromine in CHCl_3_. The spectra are dependent on the amount of used Br_2_. One Q-band with maximum at 704 nm was detected after addition of 10 μl 0.01 M Br_2_ to 2 ml of 5 × 10^−6^ M solution (molar ratio 1:10) of **4**. It is apparent that the mild oxidation changed the bisphthalocyanine molecule from a reduced form to a neutral form. With much higher Br_2_ concentration (20 μl 0.44 M, molar ratio ≈1:900) a large decrease in the Q-band intensity occurs. The Q-band is again shifted to longer wavelength and very broad peak appeared at about 750 nm.

### NIR spectroscopy

**Fig. 4:**
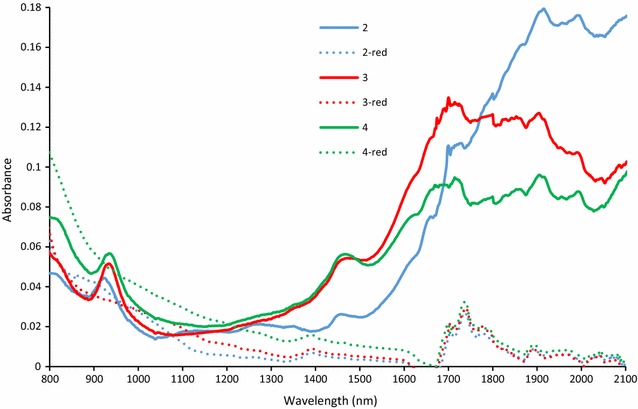
NIR spectra of reduced and neutral forms of *2*–*4* in toluene at 50 mg l^−1^

Figure 4 shows NIR spectra of reduced and neutral forms of **2**–**4** in toluene at 50 mg l^−1^. Reduced forms were formed by addition of a slight excess of triethylamine and neutral forms by addition of acetic acid. The samples were put in the dark for 24 h in order to ensure complete conversion to a desired form. The neutral forms of **2**–**4** show clearly a peak located at ~930 nm corresponding to red vibronic transition 1e_g_(π) → a_1u_(π*) from the SOMO-to-LUMO orbital [12]. The peak is very little dependent on the rare-earth metal. The second well resolved peak is at 1458–1474 nm. The most intensive signal is a broad absorption in 1600–2100 nm region, the intensity and λ_max_ is increasing with the size of the central metal.

The shape of the spectra changed completely upon reduction. The peaks characteristic for neutral form disappeared and only peaks of triethylamine at ~1400, 1700–1800 nm were observed [13].

### Acid stability

The analogous bisphthalocyanines bearing thiophene moieties have shown a very limited stability in dilute acids [9]. The next experiments were made to clarify if addition of Br as a heavy bulky substituent in 2-position on the thiophene cycle would increase acid stability. Acetic acid was chosen for stability tests due its higher compatibility with many solvents.

**Fig. 5:**
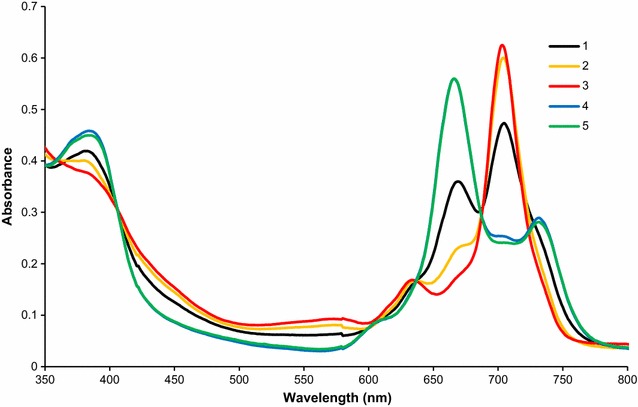
UV–vis spectra of 4 in toluene at 20 mg l^−1^ upon addition of acetic acid (AcOH). *1*: without AcOH; *2*: addition of 5 μl AcOH, reaction time 5 min; *3*: as *2*, but after 30 min; *4*: as *3*, but addition of 10 μl triethylamine (Et_3_N); *5*: control—addition of 10 μl Et_3_N to *1*

In toluene, both forms of **4** are present and it is thusly most suitable for the acid stability test. 5 microlitres of acetic acid (AcOH) was added to 2 ml toluene solution of **4** (Fig. 5). The spectra were recorded in certain time periods until constant spectra were obtained. After addition of AcOH to the sample, a decrease of the peak intensity at 660 nm was found. Proportionally, the peak at 710 nm raised by about 40%. The reaction is completed within 30 min and corresponds to the formation of a neutral form. After addition of slight excess of triethylamine (10 μl) to the neutral form, the spectrum reverts back to a reduced form (more than 95% of the initial values of curve 5 in Fig. 5). The proof that the reaction with an acid is fully reversible is indicated also by sharp isosbestic points located at 407, 636 and 687 nm, respectively.

Similar behaviour was confirmed for **2** and **3** (Additional file 3). The difference between the series lied only in the rate of conversion from the reduced to the neutral form. While the reaction for **3** and **4** is completed within 30 min, the reaction of **2** took several hours. This behaviour corresponds well with potential of first oxidation (see Table 2).

**Fig. 6:**
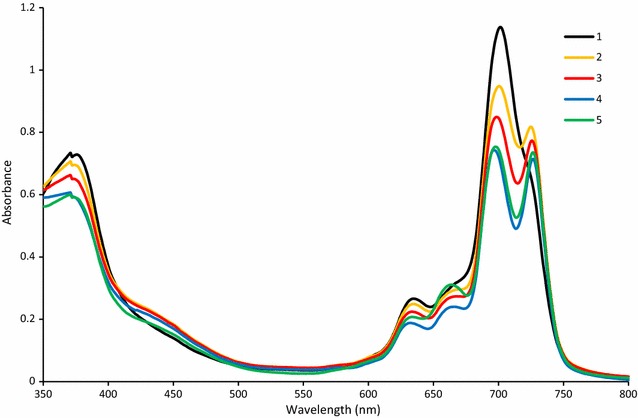
UV–vis spectra of GdPc-thiof in toluene at 20 mg l^−1^ upon addition of acetic acid (AcOH). *1*: without AcOH; *2*: addition of 5 μl AcOH, reaction time 5 min; *3*: as *2*, but after 30 min; *4*: as *2*, but after 1 h, *5*: as *4*—addition of 10 μl Et_3_N

Analogous experiment was performed with Gd analogue with non-substituted thiophene (GdPc-thiof—Fig. 6). Upon addition of AcOH totally different behaviour was found. The Q-band was splitted to two signals of nearly equal intensity indicating formation of a metal-free phthalocyanine. The full demetelation occurred in about an hour. The addition of triethylamine has no significant effect on the metal-free phthalocyanine.

From the comparison, it is apparent that the bromo substituent is sufficiently capable to stabilize the compounds effectively and confirmed our hypothesis mentioned in the introduction of the article.

### Infra-red spectroscopy

The FT-IR spectra of **2**–**4** are shown in Additional file 4. In the spectra, there are many characteristic peaks which are only minimally dependent on the rare-earth metal. The huge peak appearing at 3400–3500 cm^−1^ is O–H vibration from residual humidity present in KBr. The peaks located at about 3095, 2923 and 2852 cm^−1^ are stretching C–H vibrations of thiophene substituent at the periphery. There is no sharp peak at 2250 cm^−1^ indicating that the prepared samples were sufficiently purified from the starting nitrile. The peak at 1610 cm^−1^ is typical for phthalocyanines and corresponds to the C=C vibration of the benzene ring. The peaks at 1477, 1446, 1382, 1313, 1284, 1198, 1089, 984, 967, 902, 883, 760, 749 and 693 cm^−1^ characterize stretching and bending vibrations of benzene, pyrrole, isoindole and thiophene. The peak at 795 cm^−1^ is typical for C–Br vibration and it is shifted by 20 cm^−1^ to longer wavenumber compared to 5-methyl-2-bromothiophene [14].

### Thermogravimetry

**Fig. 7:**
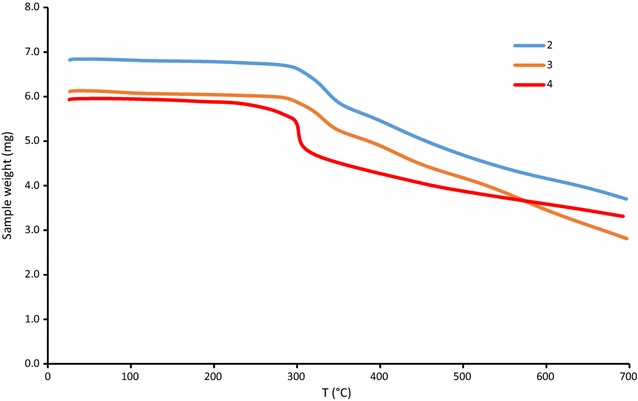
Termogravimmetric analysis of *2*–*4*

Figure 7 shows a thermal loss of **2**–**4** during heating in nitrogen atmosphere. The compounds show very similar behaviour during the heating process. The compounds are stable up to about 280 °C, then consequent slow degradation occurs. The decrease between 280 and 320 °C is more rapid for **4** then for **2** or **3**. After 320 °C the degradations have nearly the same progress for all compounds.

### Singlet oxygen production

Phthalocyanines belong to a large group of the so-called photosensitizers. Photosensitizers are materials which are capable to generate singlet oxygen (^1^O_2_) from everywhere-present triplet oxygen upon illumination with the light of suitable wavelength. The ability to generate ^1^O_2_ is characterized by singlet oxygen quantum yield Φ.

The singlet oxygen quantum yield was determined according to a reported procedure using 9,10-dimethylanthracene (DMA) [15]. The test compound was dissolved in DMF (1 mg l^−1^). The neutral form was prepared in situ by addition of diluted bromine. The decrease in absorbance was monitored using a UNICAM UV/VISIBLE Spectrophotometer, Helios Beta at 381 nm. The samples were irradiated with a red laser light (Maestro CCM, λ_max_ = 661 nm) to decrease the absorbance of DMA solution to ca. 0.2–0.3. The measurements were triplicated and no degradation of phthalocyanines during irradiation was observed. The obtained reaction half-times were corrected to the unit absorbance of the sample and related to the zinc phthalocyanine (Φ = 0.56) [16].

The estimated values of Φ for reduced and neutral forms are summarized in Table 1. The spectrum maxima for unsubstituted analogous compounds are also given. Surprisingly, Φ values for **2**–**4** are much smaller than those found for thiophene-substituted rare-earth bisphthalocyanines [9]; for compounds **2** and **3** are comparable with unsubstituted rare-earth bisphthalocyanines (Φ less than 0.01) [17]. Only **4** show some production of singlet oxygen. The difference between Φ of reduced and neutral compounds is manifested only for **4**, the value increased from 0.03 to 0.08. The oxidized forms were not measured due to a very small absorbance of oxidized state of **2**–**4** at the adopted concentration.

**Table 1:** Spectral and photochemical data for phthalocyanines **2**–**4** in DMF

Compound	Reduced form		Neutral form	Unsubstituted bisphthalocyanines	
	Q-bands (λ_max_, nm, log ε)	Φ	Φ	Q-bands (λ_max_, nm, log ε)	Φ
**2**	659 (5.41) 723 (4.95)	<0.01	<0.01	635 (4.85) 672 (4.93)	<0.01
**3**	664 (5.45) 710 (4.97)	<0.01	<0.01	628 (4.84) 671 (4.94)	<0.01
**4**	658 (5.27) 706 (5.05)	0.03 ± 0.01	0.08 ± 0.01	624 (4.55) 671 (4.67)	<0.01

### Electrochemical measurements

**Fig. 8:**
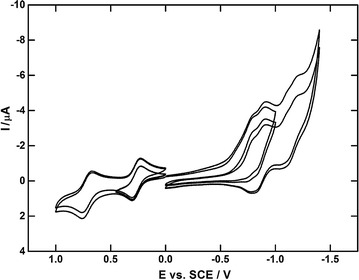
CV curves of the oxidation (reduction) of compound **3** at Pt electrode

The electrochemical characterization of described phthalocyanines was focused on first oxidation (reduction) potentials (see Table 2) reflecting the effect of metal centre as well as substitution moiety. The compounds in dichloroethane solution are likely to be in reduced form (Pc^−^). The first oxidation occurs from +0.24 to +0.32 V vs. ref yielding neutral Pc^0^. The easiest oxidation was observed for compound **4**. This is probably caused by structural effect of the Pr atom which has largest size in comparison with other two metals. In addition to this, the oxidation of all three compounds proceed in two reversible one-electron processes within the potential window. The second oxidation potential is shifted from first potential by 0.44 V to more positive values and is independent on the metal ion. When comparing oxidation potentials of presented compounds with non-brominated analogues [9], the potential of first oxidation is about 100 mV shifted towards more positive values due to electron withdrawing effect of bromo substituent (Fig. 8).

**Table 2:** Electrochemical data of **2**–**4**

Compound	E_1/2_ (ox1) (V)^a^	E_1/2_ (ox2) (V)^a^	E_1/2_ (red1) (V)^a^	E_HOMO_ (eV)^b^	E_LUMO_ (eV)^b^	ΔE (eV)^c^
**2**	0.32	0.76	−0.76	−4.72	−3.64	1.08
**3**	0.27	0.71	−0.76	−4.67	−3.64	1.03
**4**	0.24	0.68	−0.74	−4.64	−3.66	0.98

^a^E_1/2_ (ox1), E_1/2_ (ox2), E_1/2_ (red1) are half-wave potentials of the first (second) oxidation (reduction) measured by RDV

^b^E_HOMO/LUMO_ = −[E_1/2_ (ox1/red1) + 4.4] eV. All potentials are given vs. SCE

^c^ΔE = E_1/2_ (ox1) − E_1/2_ (red1), electrochemical gap

The first reduction potentials range from −0.74 to −0.76 V vs. ref., hence there are just small differences between the first reduction potentials within the series. Moreover, more reduction processes were observed but they almost merge into one. Again, when comparing first reduction potentials with previously published data [9, 18], there are not big differences, this means that variation in the substitution influences more oxidation than reduction centre.

## Conclusions

Three rare-earth metal bisphthalocyanines bearing 5-bromo-2-thienyl groups were synthesized for the first time. Their purification was achieved by flash chromatography using cellulose as an adsorbent. The prepared complexes exhibit good solubility in many organic solvents such as DMF, THF, chloroform, dichloromethane and acetone. The compounds were characterized by UV–vis, NIR, MALDI, FT-IR, thermogravimetry and elemental analysis.

Two forms of studied compounds were identified in solutions. The first form is a reduced Pc which has two maxima at 660 and 720 nm. This form has no signal in NIR area. The second form is a neutral form with one maximum located at ~700 nm. There are several characteristic peaks in NIR area. The distribution of the forms is dependent on the solvent (polarity and basicity) and the central metal. The compounds were found in reduced forms in most solvents. Transformation of the reduced form to a neutral can be achieved either by addition of small amount of acid (AcOH) or an oxidant like Br_2_. With increased concentration of Br_2_, the compounds are further oxidized to Pc^+^ and the spectra are red shifted to about 750 nm. Our hypothesis that the attachment of Br atom on the thiophene cycle should increase the acid stability was successfully confirmed. No degradation in diluted acids was found in contrary to non-brominated analogues.

Compared to thiophene-substituted rare-earth phthalocyanines a significant decrease in quantum yield of singlet oxygen Φ was found. This is in good agreement with high degree of paramagnetism found during NMR experiments. The electrochemical investigation of studied compounds has shown that the variation of central metal does not bring significant changes in the first oxidation (reduction) and HOMO (LUMO) respectively. Anyway, in comparison to previously published electrochemical data [9, 18], the substitution influences more oxidation than reduction (more HOMO than LUMO).

## Additional files

**Additional file 1.** Procedures of synthesis of **1**–**4**.

**Additional file 2.** MALDI-TOF spectra of **2**–**4**.

**Additional file 3.** UV spectra of **2** and **3** in THF and toluene.

**Additional file 4.** FT-IR spectra of **2**–**4** in KBr pellets.

## Abbreviations

UV–vis: ultraviolet–visible spectroscopy
NIR: near infra-red spectroscopy
FT-IR: Fourier transformed infra-red spectroscopy
HOMO: highest occupied molecular orbital
LUMO: lowest unoccupied molecular orbital
SOMO: single occupied molecular orbital
NMR: nuclear magnetic resonance
MALDI-TOF: matrix-assisted laser desorption/ionization time of flight mass spectrum
CV: cyclic voltammetry
RDV: rotating disk voltammetry
SCE: saturated calomel electrode
DMF: *N*,*N*-dimethylformamide
THF: tetrahydrofuran
^1^O_2_: singlet oxygen
DMA: 9,10-dimethylanthracene
Φ: quantum yield of singlet oxygen
ε: molar absorption coefficient
λ_max_: maximum wavelength of absorption
Pc: phthalocyanine

## References

[1] Kirin IS, Moskalev PN, Makashev YA (1965). Formation of phthalocyanines of rare-earth elements. Zh Neorg Khim.

[2] Simic-Glavaski B (1993). Phthalocyanine-based molecular electronic devices. Phthalocyanines.

[3] Trometer M, Even R, Simon J, Dubon A, Laval JY, Germain JP, Pauly A, Robert H (1992). Lutetium bisphthalocyanine thin films for gas detection. Sensor Actuat B Chem.

[4] Hatano M, Konami H (1991). Structures and properties of multi-layered lanthanide phthalocyanine complexes. Senryo to Yakuhin.

[5] Shirk JS, Lindle JR, Bartolli FJ, Boyle ME (1992). Third-order optical nonlinearities of bis(phthalocyanines). J Phys Chem.

[6] Ishikawa N, Sugita M, Ishikawa T, Koshihara S-Y, Kaizu Y (2003). Lanthanide double-decker complexes functioning as magnets at the single-molecular level. J Am Chem Soc.

[7] Souto J, Aroca R, DeSaja JA (1994). Gas adsorption and electrical conductivity of Langmuir–Blodgett films of terbium bisphthalocyanine. J Phys Chem.

[8] Lind SJ, Gordon KC, Gambhir S, Officer DL (2009). A spectroscopic and DFT study of thiophene-substituted metalloporphyrins as dye-sensitized solar cell dyes. Phys Chem Chem Phys.

[9] Černý J, Dokládalová L, Lyčka A, Mikysek T, Bureš F (2016). Preparation, characterization and investigation of photo-physical properties of thiophene-substituted rare-earth bisphthalocyanines. J Porphyr Phthalocyanines.

[10] Gürek AG, Ahsen V, Luneau D, Pécaut J (2001). Synthesis, structure, spectroscopic properties, and magnetic properties of an octakis(Alkylthio)-substituted lutetium(III) bisphthalocyanine. J Inorg Chem.

[11] Gürol I, Durmuş M, Ahsen V (2012). Investigation of photophysical and photochemical properties of octa-substituted double-decker rare-earth metallophthalocyanine complexes. J Porphyr Phthalocyanines.

[12] Ayhan MM, Singh A, Jeanneau E, Ahsen V, Zyss J, Ledoux-Rak I, Gürek AG, Hirel C, Bretonnière Y, Andraud C (2014). ABAB homoleptic bis(phthalocyaninato)lanthanide(III) complexes: original octupolar design leading to giant quadratic hyperpolarizability. Inorg Chem.

[13] Oliveira JIS, Pires DC, Diniz MF, Siqueira JL, Mattos EC, Rezende LC, Iha K, Dutra RCL (2014). Determination of primary amine content in bonding agent in composite solid propellants. Propell Explos Pyrot.

[14] Kamigata N, Suzuki T, Yoshida M (1990). Novel halogenation of thiophenes with benzeneseleninyl chloride and aluminium halide. Phosphorus Sulfur.

[15] Černý J, Karásková M, Rakušan J, Nešpůrek S (2010). Reactive oxygen species produced by irradiation of some phthalocyanine derivatives. J Photochem Photobiol A Chem.

[16] Lee PPS, Lo PC, Chan EYM, Fong WP, Ko WH, Ng DK (2005). Synthesis and in vitro photodynamic activity of novel galactose-containing phthalocyanines. Tetrahedron Lett.

[17] Venediktov EA (2004). Deactivation of O2 (1Δg) by diphthalocyanines of rare-earth metals. Zh Fiz Khim.

[18] Orman EF, Koca A, Özkaya AR, Gürol I, Durmuş M, Ahsen V (2014). Electrochemical, spectroelectrochemical, and electrochromic properties of lanthanide bis-phthalocyanines. J Electrochem Soc.

