# Supplementary material for: Preparation and characterization of novel double-decker rare-earth phthalocyanines substituted with 5-bromo-2-thienyl groups
Source: Chem Cent J. 2017 Apr 5;11:31. doi: 10.1186/s13065-017-0260-x (PMC5382118; doi:10.1186/s13065-017-0260-x)
Supplement: Supplementary file 1 — Additional file 1. Procedures of synthesis of 1–4. [file 13065_2017_260_MOESM1_ESM.docx]

Preparation and characterization of novel double-decker rare-earth phthalocyanines substituted with 5-bromo-2-thienyl groups

Jiří Černý, Lenka Dokládalová, Petra Horáková, Antonín Lyčka, Tomáš Mikysek, Filip Bureš

A list of additional information:

A1. Preparation of 4,5-bis(5-bromo-2-thienyl)phthalonitrile **1**

A2. Preparation of bis[octakis(5-bromo-2-thienyl)]phthalocyaninato praseodymium(III) **2**

A3. Preparation of bis[octakis(5-bromo-2-thienyl)]phthalocyaninato samarium(III) **3**

A4. Preparation of bis[octakis(5-bromo-2-thienyl)]phthalocyaninato gadolinium(III) **4**

A1. Preparation of 4,5-bis(5-bromo-2-thienyl)phthalonitrile **1**

A solution of *N*-bromosuccinimide (10.36 g, 58.23 mmol) in DMF (30 ml) was slowly added to a cold solution of 4,5-bis(2-thienyl)phthalonitrile (6.81 g, 23.29 mmol) in DMF (30 ml) under argon atmosphere. The mixture was stirred for 4.5 h in the dark. Then, the solvents were evaporated *in vacuo* and the crude product was purified by flash chromatography (SiO_2,_ CH_2_Cl_2_/hexane 2:1, then toluene and ethyl-acetate). Yield: 6.8 g (65%) of beige solid. ^1^H NMR (400 MHz, DMSO), δ (ppm) 8.42 (s, 2H), 7.36 (d, 2H), 7.31 (d, 2H). ^13^C NMR (400 MHz, DMSO), δ (ppm) 139.52, 137.16, 136.07, 131.61, 131.53, 116.07, 115.89, 114.45.

A2. Preparation of bis[octakis(5-bromo-2-thienyl)]phthalocyaninato praseodymium(III) **2**

A mixture of 4,5-bis(5-bromo-2-thienyl)phthalonitrile **1** (1.81 g, 4 mmol) and lithium (15 mg, 2.16 mmol) was refluxed for 3 h under nitrogen in anhydrous *n*-pentanol (100 ml). After cooling to about 100 °C a solution of anhydrous praseodymium acetate (165.4 mg, 0.5 mmol) in anhydrous DMF (30 ml) was added and heated to 140-145 °C for 10 h. The solvents were removed in vacuo and the resulting solid was washed with water (100 ml) and methanol (3x50 ml). Final purification was achieved by flash chromatography (cellulose, ethyl-acetate, then THF). Yield: 548 mg (29%), mp > 200 °C. Anal. calcd. for C_128_H_48_Br_16_N_16_S_16_Pr (3742.27): C 41.08; H 1.29; N 5.99; S 13.71%. Found: C 41.18; H 1.41; N 6.28; S 13.69. FT-IR: ν, cm^-1^ 3094, 2923, 2852, 2585, 2519, 1607, 1477, 1446, 1382, 1313, 1284, 1198, 1089, 1040, 984, 967, 883, 795, 760, 749. UV-VIS (DMF): λ_max_, nm (log ε) 386 (5.19), 659 (5.41), 723 (4.95). MS (MALDI-TOF (+)): *m/z* 3742.63 [M^+^], requires 3742.57.

A3. Preparation of bis[octakis(5-bromo-2-thienyl)]phthalocyaninato samarium(III) **3**

The compound was prepared in the same way as **2**, but starting with **1** (1.81 g, 4 mmol), Li (15 mg, 2.16 mmol), anhydrous samarium acetate (170.3 mg, 0.5 mmol). Yield: 258 mg (16%), mp > 200 °C. Anal. calcd. for C_128_H_48_Br_16_N_16_S_16_Sm (3751.72): C 40.98; H 1.29; N 5.97; S 13.67%. Found: C 41.08; H 1.41; N 6.29; S 13.72. FT-IR: ν, cm^-1^ 3095, 2923, 2852, 2590, 2521, 1608, 1480, 1447, 1385, 1316, 1284, 1198, 1089, 1040, 984, 967, 884, 795, 760, 750. UV-VIS (DMF): λ_max_, nm (log ε) 384 (5.22), 664 (5.45), 710 (4.97). MS (MALDI-TOF (+)): *m/z* 3753.64 [MH^+^], requires 3753.58.

A4. Preparation of bis[octakis(5-bromo-2-thienyl)]phthalocyaninato gadolinium(III) **4**

The compound was prepared in the same way as **2**, but starting with **1** (0.905 g, 2 mmol), Li (7.6 mg, 1.1 mmol), anhydrous gadolinium acetate (88 mg, 0.25 mmol). Yield: 317 mg (34%), mp > 200 °C. Anal. calcd. for C_128_H_48_Br_16_N_16_S_16_Gd (3758.61): C 40.90; H 1.29; N 5.96; S 13.65%. Found: C 40.77; H 1.54; N 5.94; S 13.79. FT-IR: ν, cm^-1^ 3096, 2925, 2853, 1611, 1483, 1445, 1387, 1321, 1285, 1199, 1089, 1042, 984, 967, 885, 796, 761, 751. UV-VIS (DMF): λ_max_, nm (log ε) 383 (5.20), 658 (5.27), 706 (5.05). MS (MALDI-TOF (+)): *m/z* 3759.65 [MH^+^], requires 3759.59.
