# Supplementary material for: Preparation and characterization of novel double-decker rare-earth phthalocyanines substituted with 5-bromo-2-thienyl groups
Source: Chem Cent J. 2017 Apr 5;11:31. doi: 10.1186/s13065-017-0260-x (PMC5382118; doi:10.1186/s13065-017-0260-x)
Supplement: Supplementary file 2 — Additional file 2. MALDI-TOF spectra of 2–4. [file 13065_2017_260_MOESM2_ESM.docx]

Preparation and characterization of novel double-decker rare-earth phthalocyanines substituted with 5-bromo-2-thienyl groups

Jiří Černý, Lenka Dokládalová, Petra Horáková, Antonín Lyčka, Tomáš Mikysek, Filip Bureš

A list of additional information:

A1. MALDI-TOF spectrum of **2**

A2. MALDI-TOF spectrum of **3**

A3. MALDI-TOF spectrum of **4**


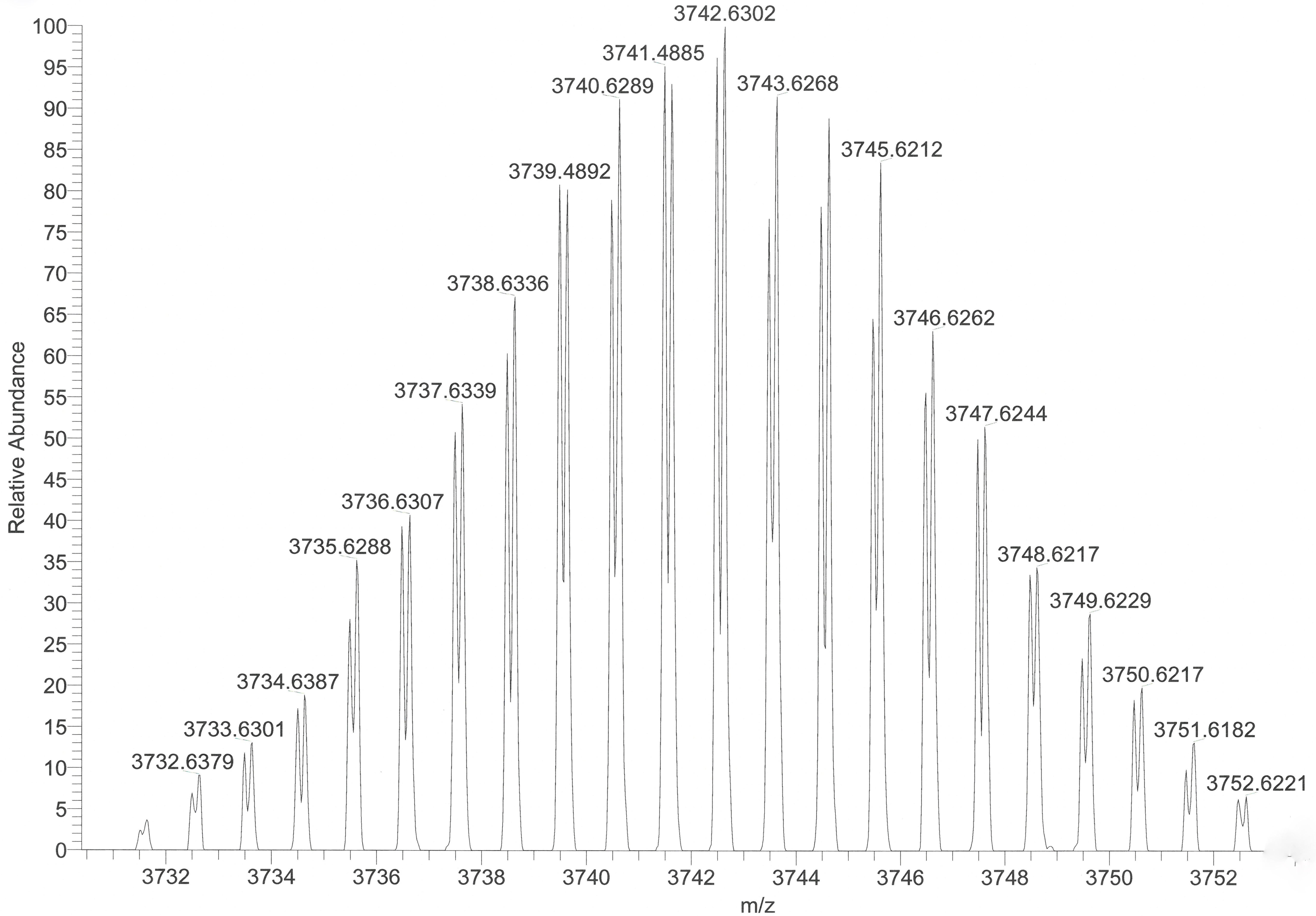


**Figure A1 MALDI-TOF spectrum of 2**


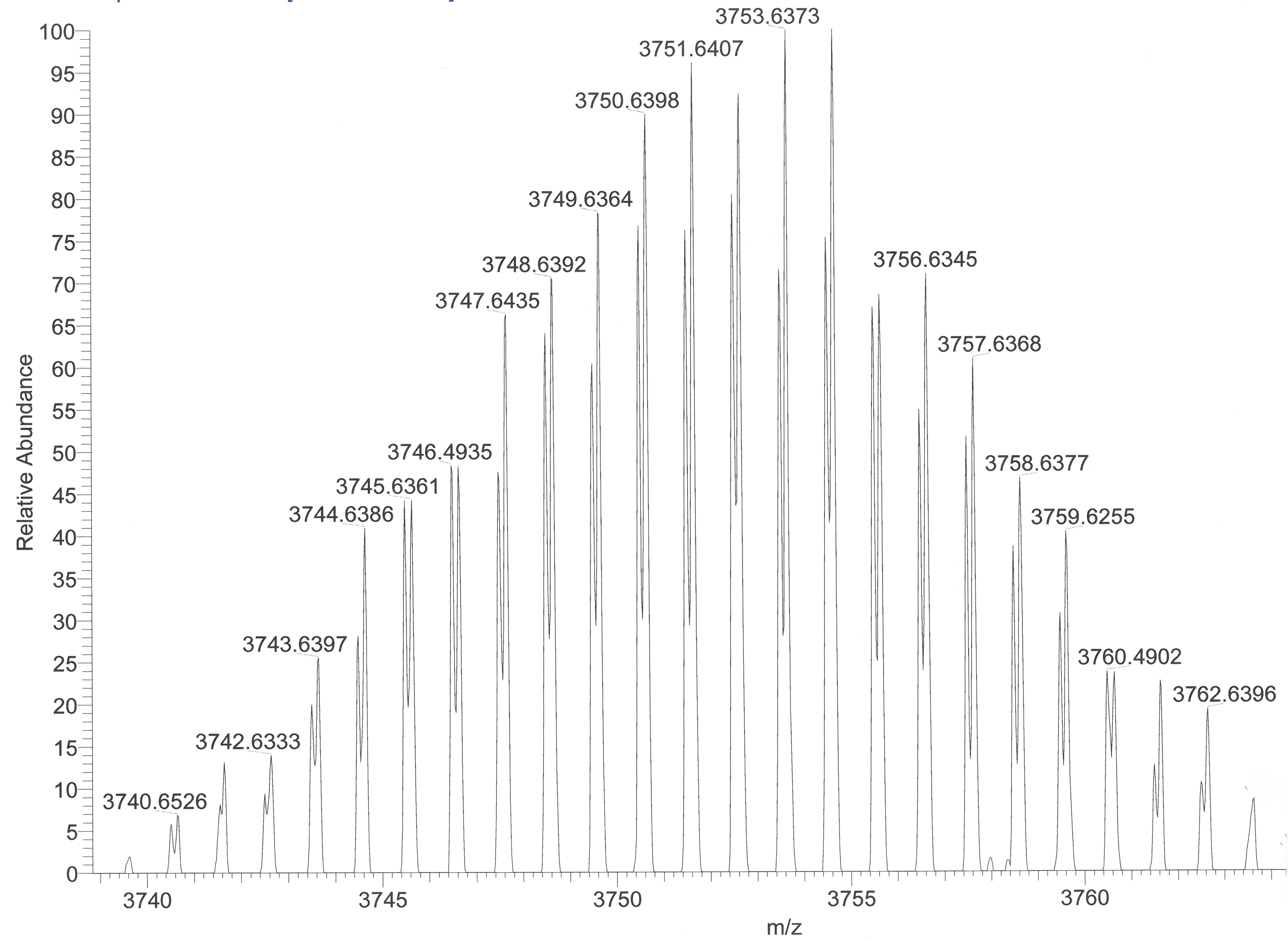


**Figure A2 MALDI-TOF spectrum of 3**


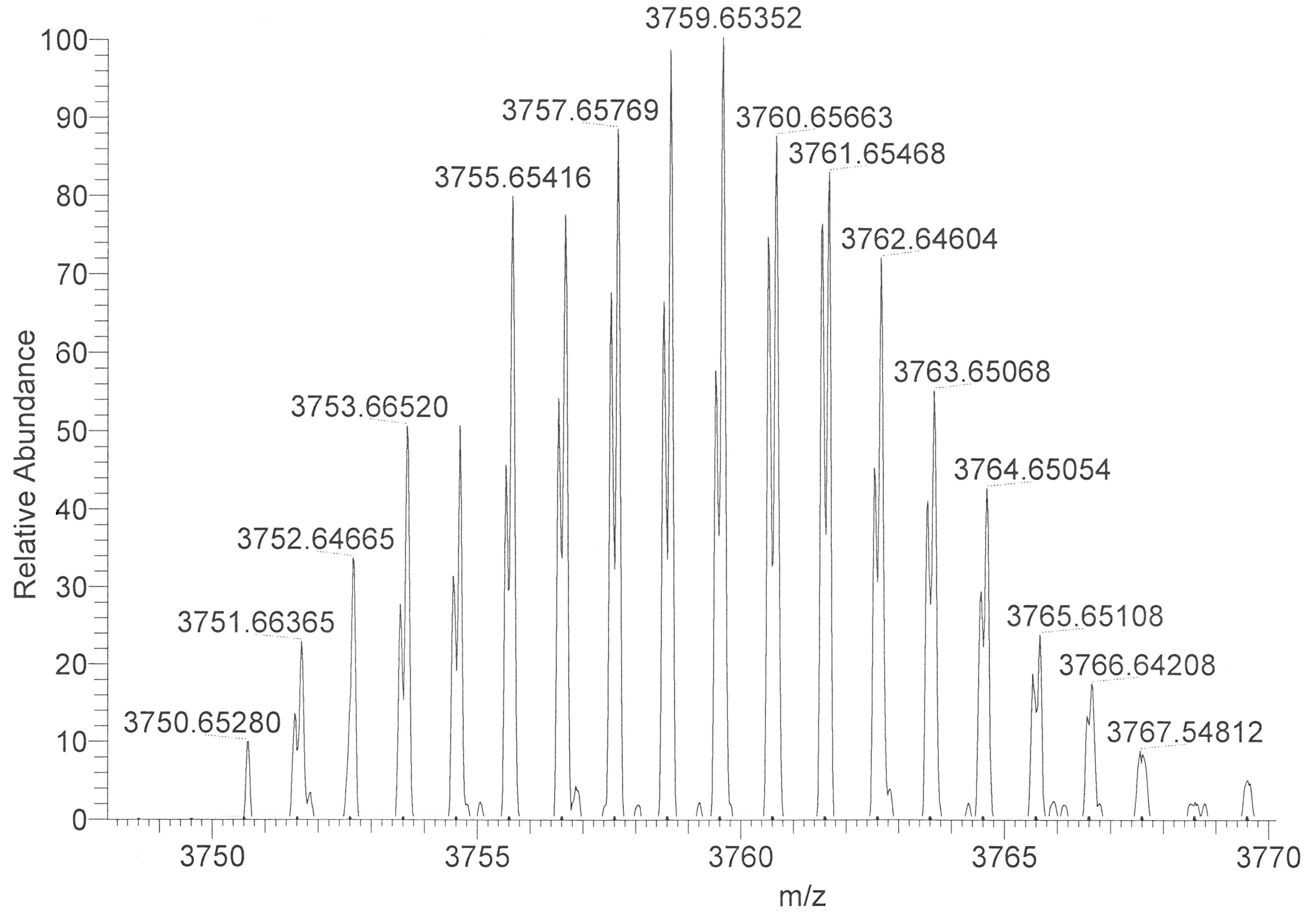


**Figure A3 MALDI-TOF spectrum of 4**
