# Supplementary material for: Preparation and characterization of novel double-decker rare-earth phthalocyanines substituted with 5-bromo-2-thienyl groups
Source: Chem Cent J. 2017 Apr 5;11:31. doi: 10.1186/s13065-017-0260-x (PMC5382118; doi:10.1186/s13065-017-0260-x)
Supplement: Supplementary file 3 — Additional file 3. UV spectra of 2 and 3 in THF and toluene. [file 13065_2017_260_MOESM3_ESM.docx]

Preparation and characterization of novel double-decker rare-earth phthalocyanines substituted with 5-bromo-2-thienyl groups

Jiří Černý, Lenka Dokládalová, Petra Horáková, Antonín Lyčka, Tomáš Mikysek, Filip Bureš

A list of additional information:

A1. UV-VIS spectra of **2** in THF and toluene

A2. UV-VIS spectra of **3** in THF

A3. UV-VIS spectra of **3** in toluene

A1. UV-VIS spectra of **2** in THF and toluene

**Figure A1.** UV-VIS spectra of **2** in THF and toluene (20 mg/l). The spectrum in toluene with acetic acid (AcOH) was recorded after 30 min after addition.

It shows that the reaction needs more time compared to **3** and **4**. This is in accordance with the values of the potential of first oxidation.

**Figure A2.** UV-VIS spectra of **3** in THF (20 mg/l).

**Figure A3.** UV-VIS spectra of **3** in toluene (20 mg/l). The spectrum with AcOH was recorded after 10 min after addition.
