# Supplementary material for: Preparation and characterization of novel double-decker rare-earth phthalocyanines substituted with 5-bromo-2-thienyl groups
Source: Chem Cent J. 2017 Apr 5;11:31. doi: 10.1186/s13065-017-0260-x (PMC5382118; doi:10.1186/s13065-017-0260-x)
Supplement: Supplementary file 4 — Additional file 4. FT-IR spectra of 2–4 in KBr pellets. [file 13065_2017_260_MOESM4_ESM.docx]

Preparation and characterization of novel double-decker rare-earth phthalocyanines substituted with 5-bromo-2-thienyl groups

Jiří Černý, Lenka Dokládalová, Petra Horáková, Antonín Lyčka, Tomáš Mikysek, Filip Bureš

A list of additional information:

A1. IR spectrum of **2**

A2. IR spectrum of **3**

A3. IR spectrum of **4**


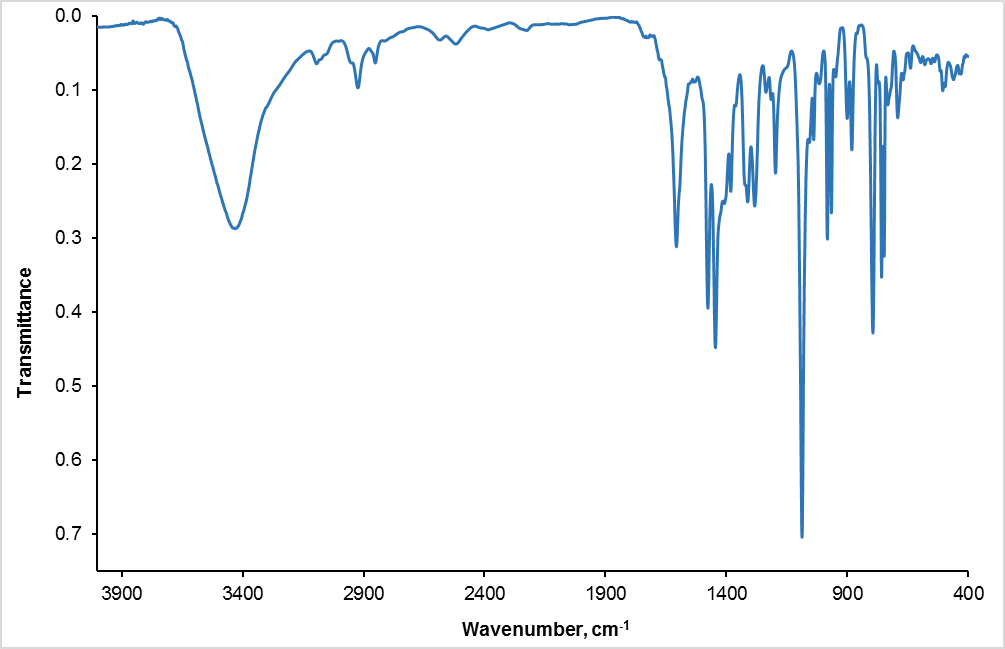


**Figure A1.** FT-IR spectrum of **2** in KBr pellet.


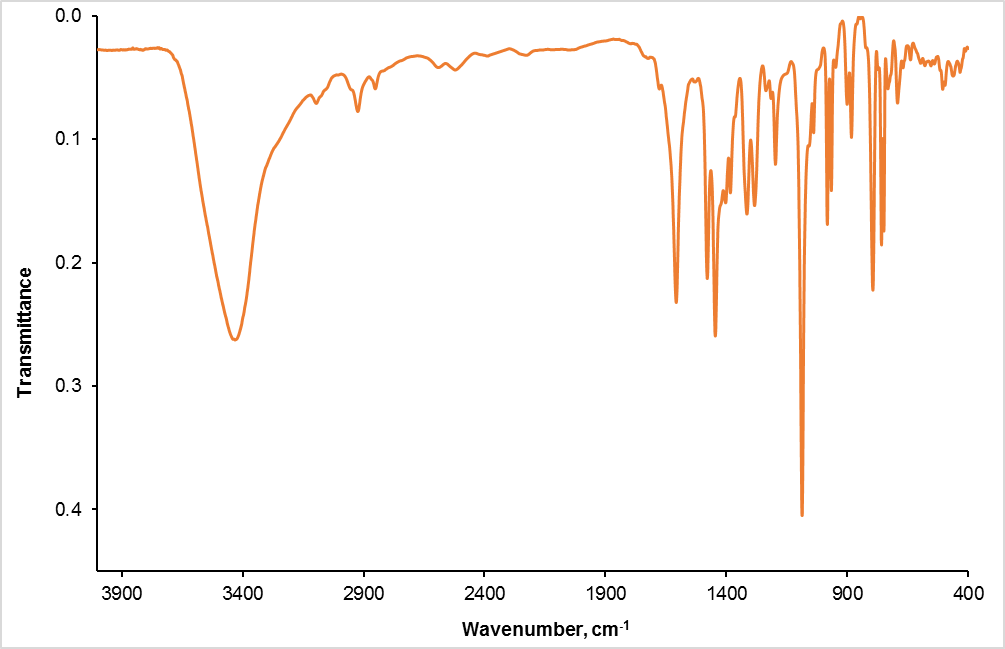


**Figure A2.** FT-IR spectrum of **3** in KBr pellet.


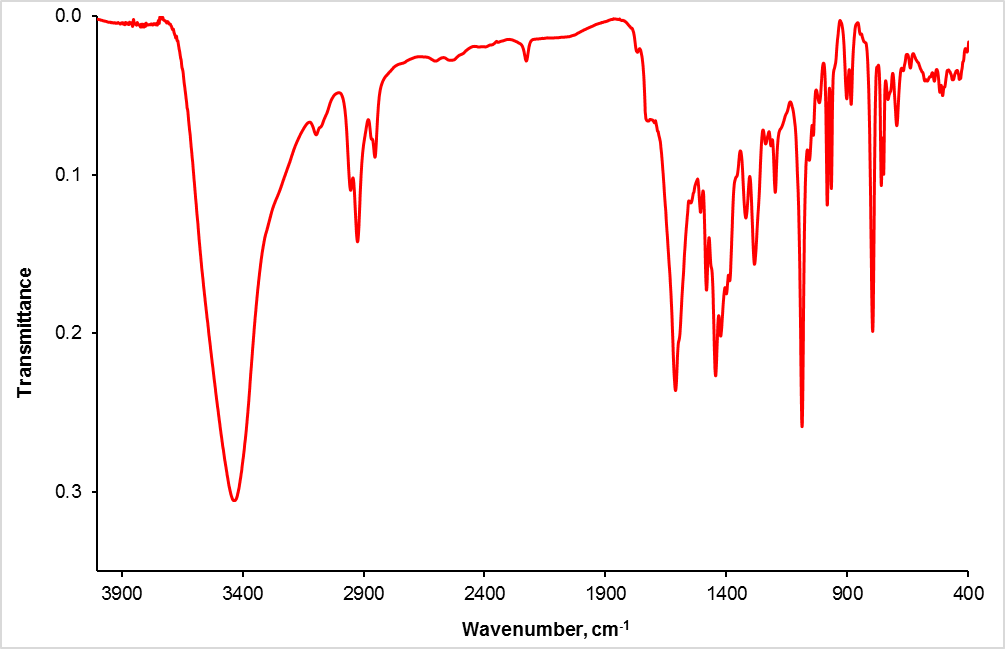


**Figure A3.** FT-IR spectrum of **4** in KBr pellet.
